# The effect of a convergent transmucosal neck on soft tissues and radiographic outcomes: a 1-year follow-up randomized controlled trial

**DOI:** 10.1007/s00784-023-04892-9

**Published:** 2023-02-07

**Authors:** Belén Morón-Conejo, Ignacio Sanz-Sánchez, Maria Paz Salido, Francisco Martínez-Rus, Guillermo Pradíes

**Affiliations:** 1 Analysis of Techniques, Material and Instruments Applied to Digital Dentistry and CAD/CAM Procedures Research Group, University Complutense, Madrid, Spain; 2 ETEP (Etiology and Therapy of Periodontal and Peri-Implant Diseases) Research Group, University Complutense, Madrid, Spain; 3 Department of Conservative and Prosthetic Dentistry, Faculty of Dentistry, University Complutense of Madrid, Plaza Ramón Y Cajal S/N. 28040, Madrid, Spain

**Keywords:** Convergent neck, Dental implant, Soft tissues, Bone loss, Volumetric analysis

## Abstract

**Purpose:**

The aim of this randomized controlled clinical trial was to evaluate peri-implant marginal bone levels (MBLs) and soft tissue dimension changes 1 year after loading. Patients in the control group received bone-level implants, whereas in the test group, tissue-level implants with a convergent transmucosal neck were used.

**Material and methods:**

MBLs were calculated by measuring the distance from the implant shoulder to the first visible bone-to-implant contact using standardized periapical digital radiographs. Baseline (day of loading) and follow-up digital models obtained with an intraoral scanner were used to quantify the changes in the peri-implant soft tissue dimensions with a best-fit algorithm.

**Results:**

The difference between final and baseline MBLs showed a mean bone loss of 0.16 ± 0.01 mm in the test group (*n* = 15) and 0.45 ± 0.09 mm in the control group (*n* = 14) (*p* > 0.05). Soft tissue contour at the level of the gingival margin (GM) increased by 1.96 ± 2.69 mm in the test group and 0.65 ± 0.42 mm in the control group (*p* = 0.167). Both groups showed a coronal displacement of the gingival margin with no significant differences among them.

**Conclusions:**

The present study demonstrated peri-implant hard and soft tissues stability at both implant designs with no significant differences 12 months after loading.

**Clinical relevance:**

There is still insufficient scientific evidence to demonstrate the role and advantages of the convergent transmucosal neck on the behavior of the peri-implant soft and hard tissues stability compared to a straight neck in bone-level implants 12 months after loading.

## Introduction

Implant-supported restorations to rehabilitate partially edentulous patients have become a standardized and predictable therapy with high survival and success rates [1, 2]. Several factors have shown to influence peri-implant hard and soft tissues stability, such as the implant macro-morphology (thread, body shape, and neck design), the surface micro-topography, or the implant-abutment connection [3–5]. Today, there are two main types of implants on the market depending on their relationship with soft and hard tissues, bone-level and tissue-level implants. These implants have different neck configurations and prosthetic abutment connections that have been the subject of study for their possible relationship with soft and hard tissue stability [4, 5]. The implant neck configuration is a crucial area of any endosseous implant [4]. Bone-level implants are those in which the entire implant surface is completely inserted into the bone. On the contrary, tissue-level implants present a transmucosal neck that is left in contact with the soft tissues, either with a straight or divergent configuration [4]. The most common configuration for tissue-level implants is a divergent tulip-shaped transmucosal neck that has demonstrated predictable results with a decrease in inflammation and bone loss, although it might have certain disadvantages on esthetic outcomes due to the space reduction for soft tissues [4, 5].

The Biologically Oriented Dental Preparation Technique (B.O.P.T.) has been proposed as a technique to vertically prepare dental abutments without a finishing line [6]. This preparation technique involves an intrasulcular preparation of the tooth without a finishing line and the use of a temporary restoration that allows the stabilization of the clot at that level. During tooth preparation, previous finishing lines are eliminated, and the convexity of the crown is reduced, so a full continuity with the tooth root is created [6]. The temporary crown guides the healing and reinsertion of the soft tissues with its prosthetic profile. After at least 1 month of stabilization of the soft tissues around the new emergence profile formed with the temporary restorations, impressions are taken, and the definitive restoration is fabricated according to a defined laboratory protocol. The creation of a new emergence profile with the restoration allows the soft tissue to adapt to the new profile, increasing its thickness and, thus, the stability of the gingival margin [6–9].

These concepts have been transferred to implant-supported restorations by designing an implant with a convergent transmucosal neck and abutments without a finishing line (Prama, Sweden & Martina). This concept is in line with a published guideline for the management of the implant restoration, where a subcritical contour as convergent (concave) as possible is recommended [10]. The convergent transmucosal neck creates space for the soft tissues and provides a regenerative space that leads to a stable coagulum, which potentially will turn into soft tissue, increasing the peri-implant tissue thickness [11]. The convergent transmucosal neck has also shown advantages in terms of peri-implant hard tissues. Furthermore, some studies reported less peri-implant marginal bone loss around implants with a convergent transmucosal neck as compared to a divergent neck [12, 13]. Nevertheless, there is still insufficient scientific evidence to demonstrate the role of the neck morphology on the behavior of the peri-implant soft tissue.

Today, there is a vast variety of tools and techniques to assess the dimensions and the appearance of peri-implant soft tissues. Several indices have been proposed, such as the pink esthetic score (PES) (14) or the PES and white esthetic score (PES/WES) [15]. Also, horizontal probing with a periodontal probe or with an endodontic file has been used [8, 16]. With the advent of digital dentistry, a new software has been developed to accurately evaluate dimensional changes of the hard and soft tissues [17–19]. Basically, the software is used to superimpose two files obtained with an intra- or extra-oral scanner using a best-fit algorithm [20]. The differences in distance and volume between both casts can be analyzed graphically with a color code and quantitatively in millimeters, with very low errors between 1 and 10 µm [21–23].

**Fig. 1:**
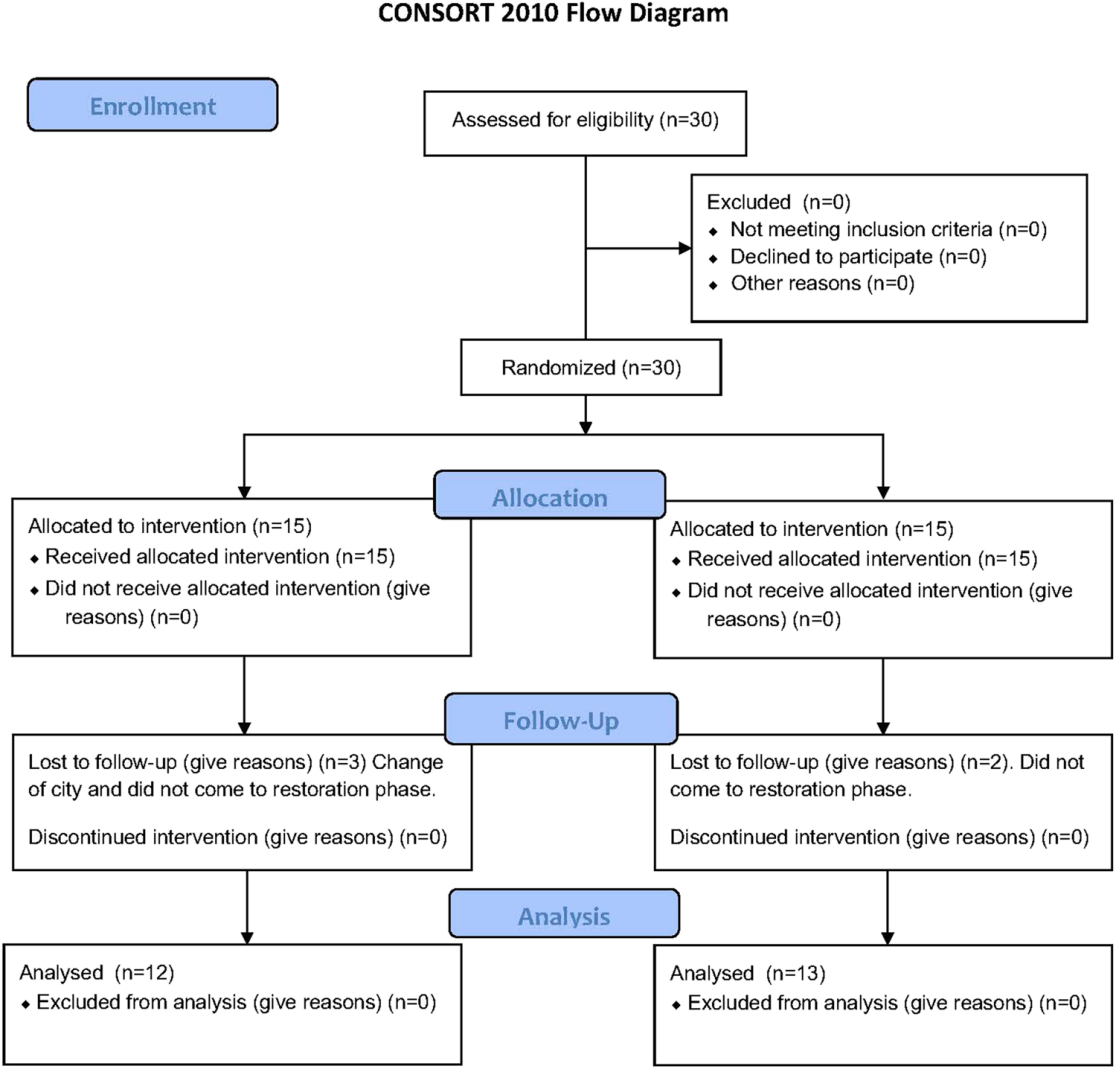
CONSORT flow diagram

Therefore, the aim of the present randomized clinical trial was to evaluate the efficacy of tissue-level implants with a convergent transmucosal neck and abutments without a finishing line on marginal bone levels (MBLs) and soft tissue dimensions changes 12 months after loading and compare it to conventional bone-level implants with abutments with a horizontal finishing line (Fig. 1).

## Material and methods

### Study design

The study was designed as a randomized controlled clinical study with a parallel group design and a 12-month follow-up. The proposed null hypothesis was that there would not be differences in terms of MBL changes or soft tissue dimensions changes between two implants with different neck configurations 1 year after loading. The clinical study protocol was approved by the Ethics Committee of the Hospital Clinic San Carlos in Madrid (C.P.-C.I. 14/532-P) and followed the ethical principles founded in the Declaration of Helsinki. The protocol was registered in ClinicalTrials.gov (identifier NCT05051839). All patients gave their written informed consent and accepted to take part of the clinical trial.

### Subject population

Consecutive patients were selected from those attending the Postgraduate Clinic in Restorative Dentistry based in the New Technologies, University Complutense of Madrid. The screening examination included the following:

- Cone-beam CT to determine the bone availability for dental implants.
- Clinical examination to evaluate the patient inclusion and exclusion criteria.
- Standardized intraoral photographs for registering the baseline site status.

Patients were selected based on the following criteria.

- Inclusion criteria
  - Male or female ≥ 18 years old.
  - Need for implant-supported fixed prosthesis in the posterior area of the upper or lower jaw (up to 2 implants and three units bridge). Free-ended situations without a distal tooth were allowed.
  - Bone quantity at the implant site to allow the insertion of Sweden and Martina Premium or Prama implants (Due Carrare, Padova, Italy) with diameters between 3.8 and 5 mm (mm) and lengths between 10 and 13 mm.
  - Healthy ASA type I and II patients.
  - Full-mouth plaque index < 20% (percentage of sites with plaque measured at 6 sites per tooth in every tooth of the mouth).
  - Presence of ≥ 2 mm of keratinized mucosa at the buccal and lingual portion of the implant.
- Exclusion criteria
  - Smokers ≥ 10 cigarettes/day.
  - Presence of implant-supported restorations adjacent to the study site.
  - Active periodontitis defined as the presence of pockets with probing depth (PD) ≥ 5 mm and bleeding on probing (BoP).
  - Systemic medication that contraindicates surgery (bisphosphonates or steroid therapy).
  - Uncontrolled diabetes (HbA1c > 7.5).
  - Severe bruxism.
  - Pregnancy.
  - Previous history of radiotherapy.
- Exclusion criteria at surgery
  - Vertical soft tissue thickness < 2 mm assessed with a periodontal probe after placing anesthesia at the mid crestal portion of the edentulous site.
  - Lack of primary stability defined as < 10 Ncm measured by hand torque wrench.
  - Need of augmentation procedures in the presence of dehiscences or fenestrations.
  - Unable to place the implant according to the prosthetic requirements.

### Study groups

**Fig. 2:**
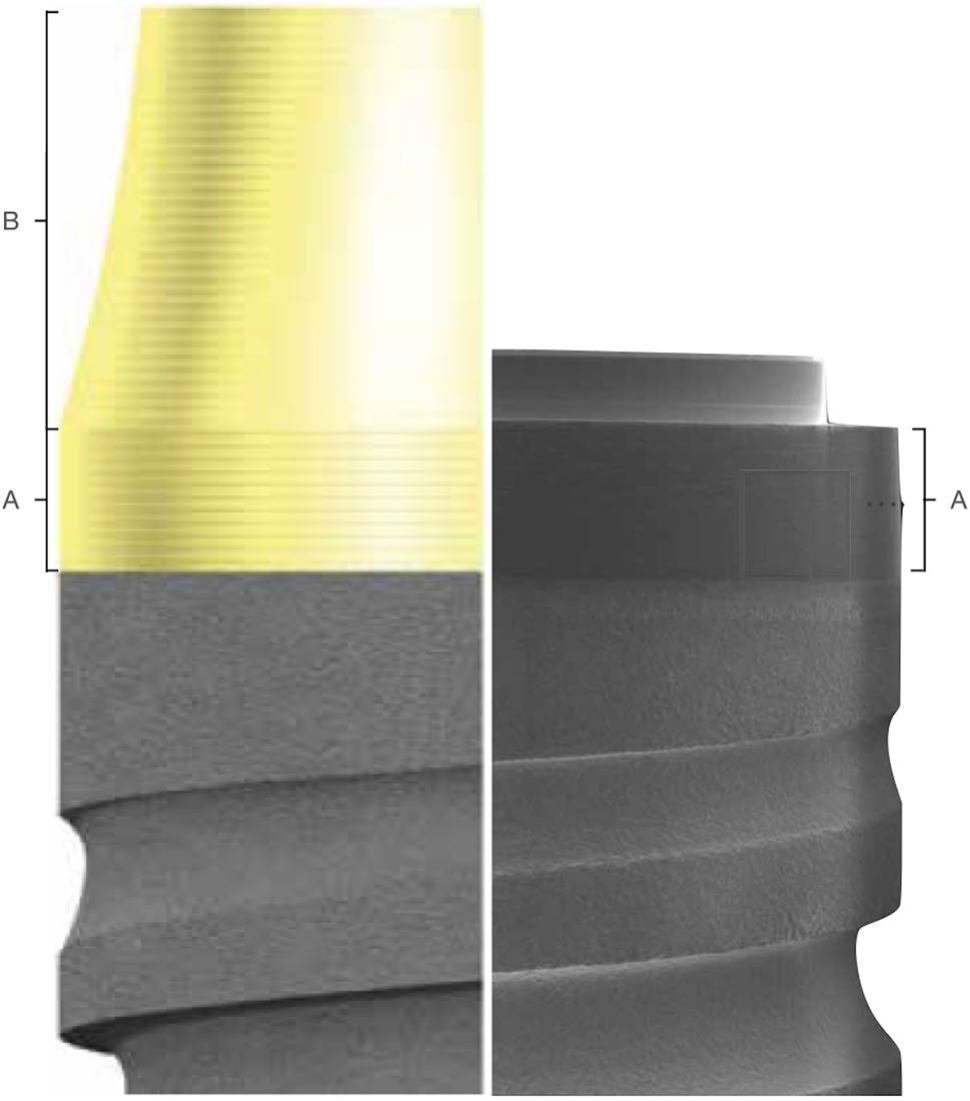
Test and control group implant design. In the left, the test group (Prama, Sweden and Martina), and in the right, the control group (Premium, Sweden and Martina). The implant design characteristics: **A** 0.8 mm straight micro-threaded neck. **B** 2.00 mm convergent micro-threaded neck

Figure 2 depicts the characteristics of the study groups.

#### Control group (*n* = 19)

Patients in the control group received bone-level implants with a 0.8 mm height straight and machined rim and a 3.4 mm internal hex prosthetic connection (Premium SP, Sweden & Martina). In the restoration phase, titanium abutments with a straight emergence from the implant head and a horizontal finish line were used.

#### Test group (*n* = 19)

Patients in the test group received tissue-level implants with a 2.8 mm convergent transmucosal neck and an ultrathin threaded microsurface (UTM). The first portion consisted of a 0.8 mm height straight neck followed by a hyperbolic convergent portion of 2 mm height. The prosthetic connection was an internal hex of 3.4 mm (Prama, Sweden & Martina). In the restoration phase, titanium abutments without a finish line and a continuous surface with the convergent transmucosal neck were used following the B.O.P.T. philosophy.

### Surgical phase

**Fig. 3:**
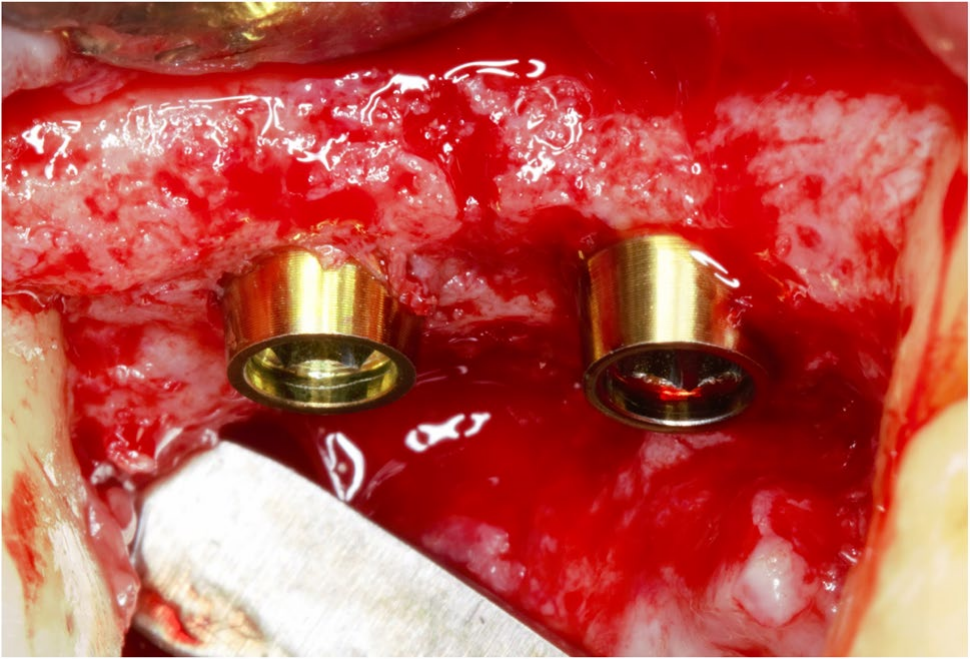
Convergent transmucosal neck implant placement surgery. Test group. (Prama, Sweden and Martina, Italy)

All surgeries were performed by two experienced and calibrated specialists in dental implantology (GP and IS). All patients followed the same surgical protocol under local anesthesia with articaine 4% and adrenalin (1:100.000). A mid-supracrestal incision was made including the adjacent teeth, and a full-thickness mucoperiosteal flap was raised. Implant sites were prepared following the manufacturer’s instructions. Sealed envelopes were opened. The implant insertion was the same for both the test and control groups. The 0.8 mm straight section of the machined rim in the control implants and the ultrathin threaded microsurface in the test implants were submerged at the level of the crestal bone. In the test group, the hyperbolic neck portion was not submerged (Fig. 3). Implant diameter was selected to allow at least 1.5 mm of buccal and lingual bone. In the case of multiple implants, a distance ≥ 3 mm between implants was assured. A non-submerged protocol was used, and the flaps were replaced and sutured around the corresponding straight healing abutments.

### Restorative phase

**Fig. 4:**
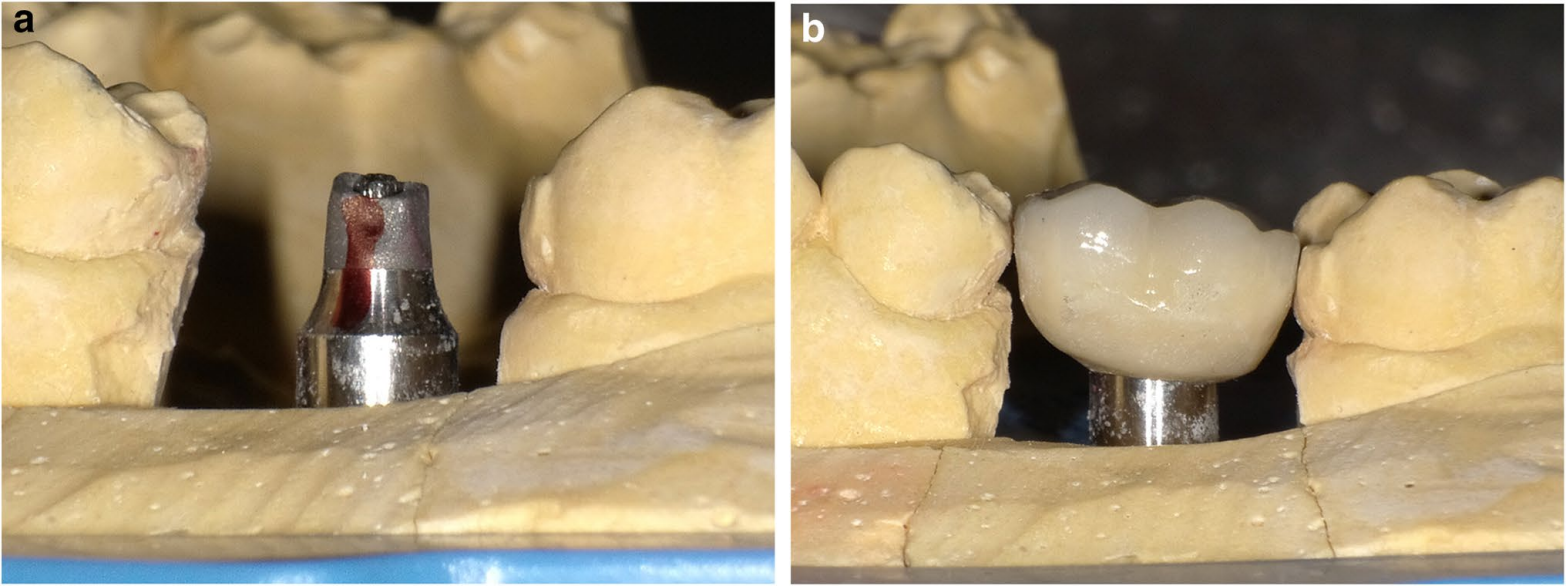
Test group final restorations: **A** Final cast with Prama (Sweden and Martina, Italy) abutments without finishing line. **B** Fused metal restorations for Prama implants (Sweden and Martina, Italy)

**Fig. 5:**
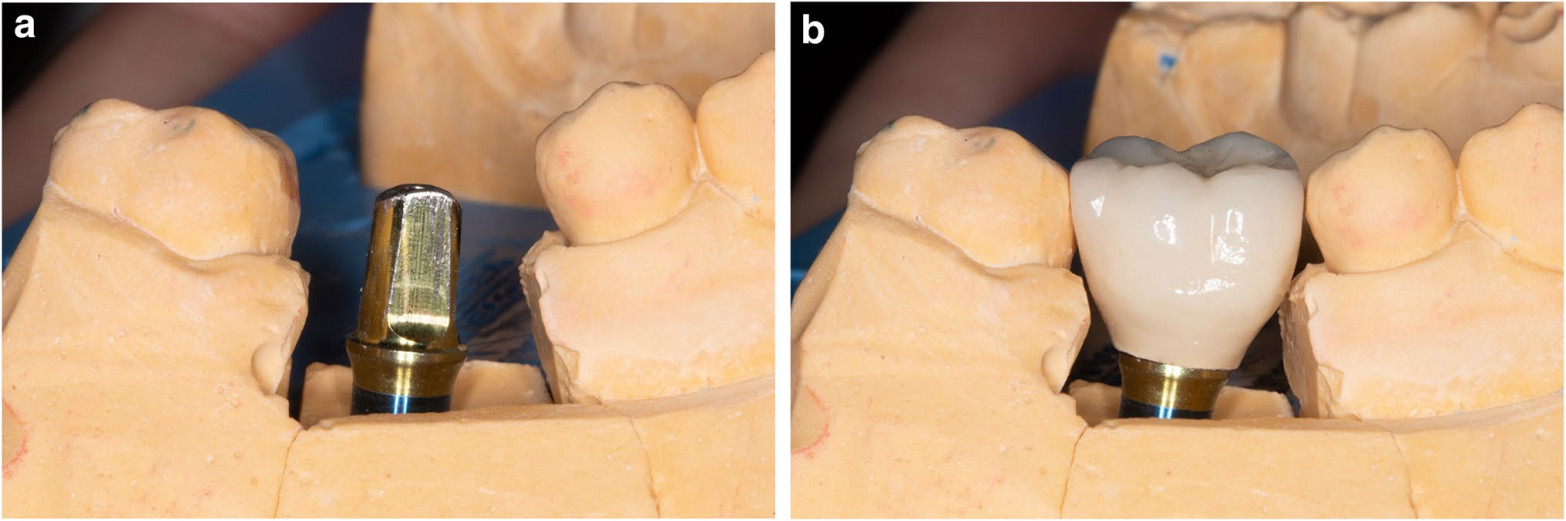
Control group final restoration: **A** Final cast with Premium (Sweden and Martina, Italy) abutments with horizontal termination line. **B** Fused to metal restoration for Premium implants (Sweden and Martina, Italy)

Twelve weeks after surgery**,** impressions were taken at the implant level with an elastomer material and an open tray technique. The final fused to metal restorations were cemented to the prosthetic implant abutment with a resin cement (Stone Implant, Sweden & Martina), and care was placed to remove any excess of cement. In the test group, titanium abutments without a finish line (continuous surface with the convergent transmucosal neck) were used following the B.O.P.T. philosophy (Fig. 4). The restorations were fabricated with a slight inclusion in the mucosal sulcus of approximately 1 mm and an emergence profile of 45° [6]. In the control group, titanium abutments with a horizontal finishing line were used. These abutments presented a straight emergence and a fixed horizontal limit on which the metal and ceramic structure support (Fig. 5). None of the implants apply the concept of platform switching. After functional loading, periapical radiographs using an individualized film-holder (Dentsply) were taken to record MBLs, and digital models were obtained with an intraoral scanner (Cerec Omnicam, Denstply Sirona, Germany). The day of loading was considered for baseline measurements. Each patient was enrolled in an annual follow-up program where outcomes were registered and periodontal and peri-implant maintenance therapy was provided by means of ultrasound and an air-polishing device (Airflow® Prophylaxis Master, EMS®, Nyon, Switzerland).

### Measurements and outcome variables

**Fig. 6:**
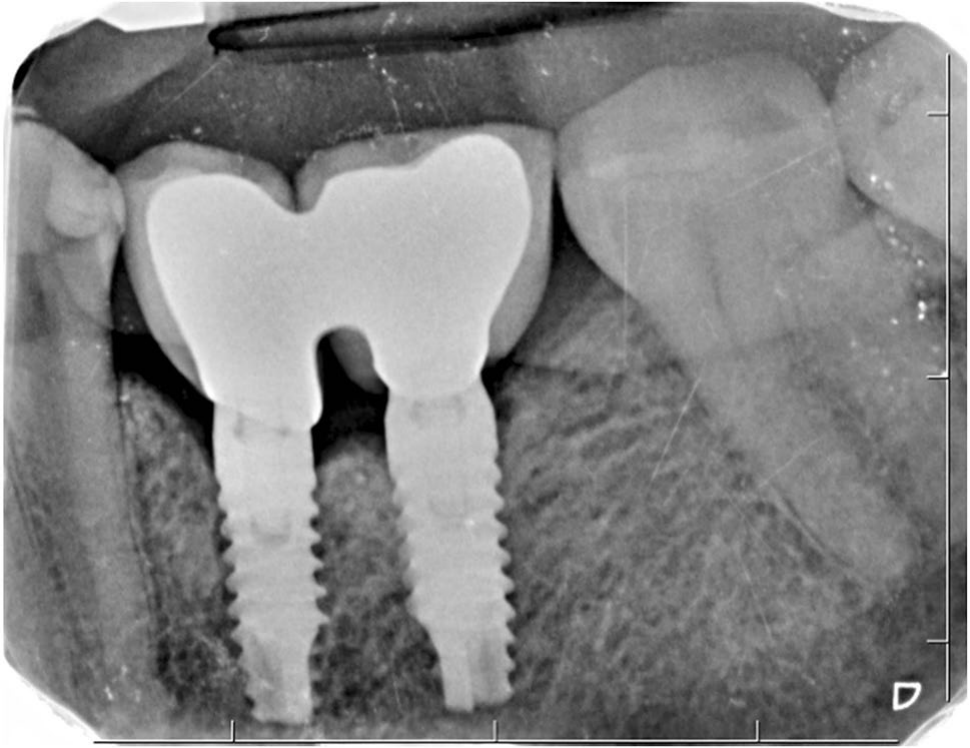
Baseline standardized periapical radiograph on the day of cementation in the study group

The primary outcome of the present RCT was the change on MBLs between baseline and 12 months. MBLs were calculated by measuring the distance from the implant shoulder to the first visible bone-to-implant contact at the mesial and distal aspect of each implant (Fig. 6). The standardization of the radiograph was achieved using a parallel technique with the aid of Rinn holders and individual silicon bite registrations. One calibrated examiner executed all the measurements by means of computer image analysis software (Image J. National Institutes of Health [NIH], Bethesda, MD, USA). Intra-examiner reliability was assessed by means of a calibrating session where 15 random radiographs were measured twice (kappa values > 0.8). The elimination of image distortions and the determination of the exact magnification were achieved by calibrating all images using the known distance between two implant threads and the length of the implants. Positive values for these changes indicate bone loss. The average between the mesial and distal measurements was calculated. Due to the different macroscopic designs of both implants, the examiner could not be blinded.

#### Secondary outcomes

##### Linear soft tissues dimensions

**Fig. 7:**
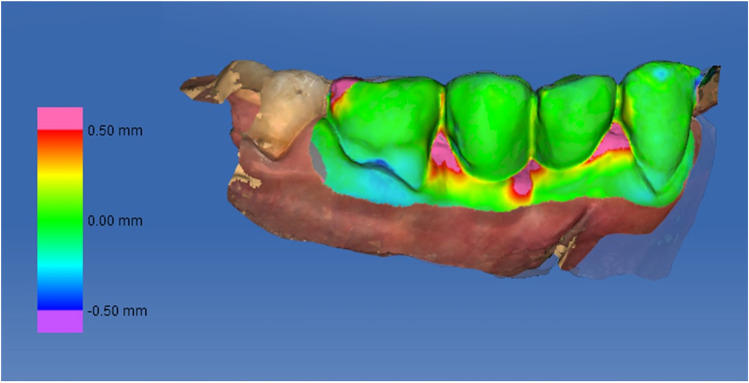
Graphic image of the analysis of the distance between the digital casts with the color code in a case of the study group (OraCheck, Cyfex, Switzerland)

**Fig. 8:**
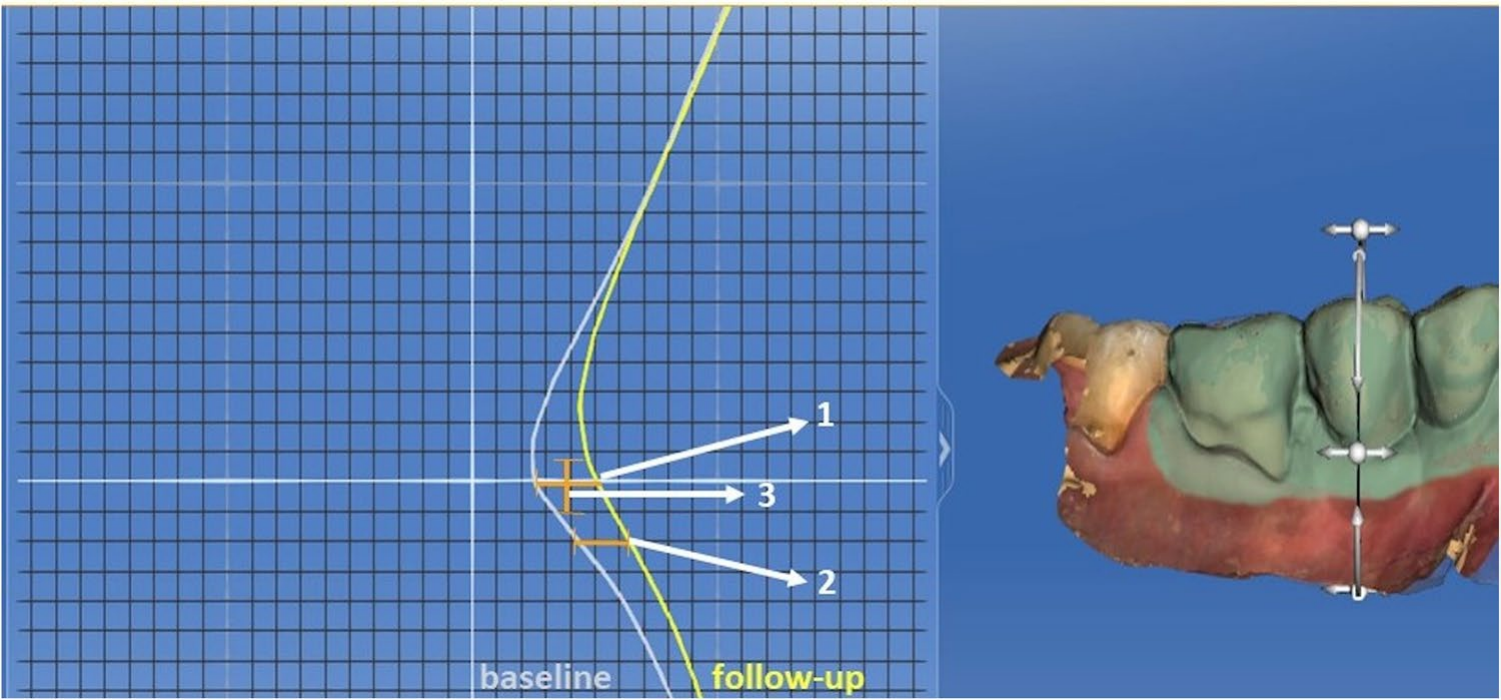
Diagram of the horizontal measurements: (1) Horizontal measurement from the basal gingival margin at the same height in the follow-up scan (difference in thickness at the gingival margin). (2) Horizontal measurement 2 mm apical from the baseline gingival margin at the same height in the follow-up scan (difference in thickness at 2 mm from gingival margin). (3) Vertical measurement from the inflection point between the crown curvature and the peri-implant soft tissue to that same point of the follow-up scan (height difference of the gingival margin) (OraCheck, Cyfex, Switzerland)

The soft tissues contour measurements were performed using digital impressions taken at baseline and at the 12-month follow-up visit, which were uploaded into a software (OraCheck, Cyfex, Switzerland) to analyze the changes in the soft tissue dimensions. Both files were superimposed using a best-fit algorithm to obtain an overlap with the smallest possible discrepancy. The implant-supported restorations, the adjacent mesial and distal teeth, and its keratinized gingiva were selected for the analysis with the selection tool (Fig. 7). Linear measurements were obtained from a cross-sectional plane placing a transversal cut line in the center of the clinical crown and following the central axis of the implant [18]. The crowns should have both lines overlapping as there should be no difference between them (if the best-fit algorithm was carried out properly). The images were enlarged on a 1 × 1 mm grid, and four measurements were performed by one blinded and calibrated examiner at the buccal and lingual surfaces (Fig. 8):

1. Horizontal linear measurement at the level of the initial mucosal margin. The difference between both measurements represents the change in contour thickness at the most coronal portion.
2. Horizontal linear measurement 2 mm apical to the initial mucosal margin. The difference between both measurements represents the change in contour thickness 2 mm apical to the position of the mucosal margin at baseline.
3. Mucosal recession was assessed by measuring the vertical distance between the position of the mucosal margin at the different time points.
4. The volume was analyzed with a color-coded graph in µm, which corresponds to the mean distance between the two surfaces within the analyzed area of interest (implant and adjacent teeth). The yellow, orange, red, and pink colors represent an increased distance, which is equivalent to an increase in tissue contour thickness. Those areas in blue and violet color indicated a loss in thickness. A color-coded scale ranging from + 500 to − 500 µm was selected.

##### Peri-implantitis

It is defined as the presence of BOP and/or suppuration together with increased probing pocket depth and radiographic bone loss > 2 mm during the first year after loading [24]. The incidence of peri-implantitis was calculated at 12 months after loading.

### Statistical analysis

The sample size was calculated for detecting a mean difference between test and control groups in radiographic marginal bone level changes of 1 mm with a standard deviation (SD) of 1.25 mm, an alpha error of 0.05, and a statistical power of 80% (G*Power 3.1 Software). After a pilot study with 10 patients comparing both groups with a mean marginal bone loss in the test group of 1.25 ± 1.25 mm and in the control group of 2.37 ± 0.69 mm, the resulting sample size was 28. Each patient was randomized to receive one of the two types of implants by means of a computer-generated random list and by blocks of four treatments each. Allocation concealment was assured using opaque sealed envelopes that were opened immediately after implant site preparation.

Descriptive statistics (mean values, standard deviations, medians, and 95% confidence intervals) of variables were performed for each group using a statistical software program (SPSS version 25.00; IBM, USA). The implant was considered the unit of analysis. The normality was assessed by means of a Kolmogorov–Smirnov test and was found to be normally distributed. The *t*-test for independent samples was carried out to compare the continuous outcomes between the test and control groups. Pearson’s correlation coefficient was applied to measure the possible association between the marginal bone loss and the gingival margin level. The alpha level (type I error) was set at 0.05, and the implant was considered the statistical unit.

## Results

### Patient population and follow-up

Twenty-five patients with 38 implants were included in the study and received surgery (Table 1). At the end of the follow-up period, the actual sample size was 25 patients with 34 implants who completed the study. The reasons for patient withdrawal are depicted in Fig. 1. Implant survival was 100%, and all surgeries healed uneventfully.

**Table 1:** Baseline demographic characteristics of the study sample

Implants							
	Total implants (*n* = 38)	Total subject (*n* = 25)	Control implants (*n* = 19)	Control subjects (*n* = 13)	Test implants (*n* = 19)	Test subjects (*n* = 12)	*P*-value
Sex (*n* and %)							
Male	13	52%	7	53.84%	6	50%	0.841
Female	12	48%	6	46.16%	6	50%	
Age (mean and SD)	44.52		43.68		48.68		0.881
Smoking (*n* and %)							
Non-smoker	20	80%	11	84.61%	9	75%	**0.003**
Former smoker	5	20%	2	15.39%	3	25%	
Number of implant placed							
Single units	16	42.10%	9	47.36%	7	36.84%	0.052
Multiple units	22	57.89%	10	52.63%	12	63.15%	
Implants position							
Premolars	11	28.94%	4	21.05%	7	36.84%	** < 0.001**
Molars	27	71.05%	15	78.94%	12	63.15%	
Implant diameter							
3.8 mm	29	76.31%	14	73.68%	15	78.94%	** < 0.001**
4.25 mm	8	21.05%	5	26.31%	3	15.78%	
5 mm	1	2.63%	0	0%	1	5.26%	
Implant length							
10 mm	18	47.36%	8	42.10%	10	52.36%	**0.004**
11.5 mm	17	44.73%	9	47.36%	8	42.10%	
13 mm	3	7.89%	2	10.52%	1	5.26%	

### Marginal bone level changes

Table 2 depicts the mean changes in MBLS at the mesial, distal, and both sites within each group. In general, bone loss was low, and no significant differences between groups could be detected. Mean bone loss was 0.16 ± 0.01 mm in the test group and 0.45 ± 0.09 mm in the control group (*p* = 0.484). At the end of the follow-up period, 3 implants in the test (15.78%) and 1 implant in the control group presented peri-implantitis (5.25%). The chi-square test showed no statistically significant differences between the two groups in terms of peri-implantitis (*p* = 0.347).

**Table 2:** Marginal bone level changes at 12-month follow-up at millimeters (mm)

	Mesial	Distal	Mean
Test group	0.16 ± 0.92	0.17 ± 0.91	0.165 ± 0.007
Control group	0.52 ± 1.46	0.39 ± 1.18	0.455 ± 0.091
*p*-value	0.460	0.587	0.484

### Linear soft tissues contour measurements

The linear increase in buccal soft tissue contour thickness at the level of the mucosal margin was greater in the test (1.96 ± 2.69 mm) than in the control group (0.65 ± 0.42 mm), although this difference was not statistically significant. Two mm apically to the buccal mucosal margin, the increase was minimal and similar in both groups (Table 3). At 12 months, the test implants showed a coronal displacement of the buccal mucosal margin of 0.37 ± 0.40 mm, whereas in the control implant, this value was 0.28 ± 0.29 mm. Table 3 depicts the behavior of the soft tissue contour at the lingual aspect of the implant, where no significant differences could be observed for any of the studied parameters, although the linear increase in lingual soft tissue contour thickness at the level of the mucosal margin was greater in control (1.58 ± 2.804 mm) than in the test group (0.58 ± 0.37 mm). The correlation between the MBL and the level of the mucosal margin at the 12-month follow-up was calculated, and it was not statistically significant.

**Table 3:** Linear soft tissues contour changes at 12-month follow-up in millimeters. (GM, gingival margin)

	Control group	Test group	*p*-values
Thickness change at the buccal GM	0.646 ± 0.422	1.963 ± 2.693	0.167
Thickness change 2 mm apically to the buccal GM	0.156 ± 0.241	0.147 ± 0.246	0.943
Vertical difference at the buccal GM	0.283 ± 0.296	0.372 ± 0.40	0.600
Thickness change at the lingual GM	1.580 ± 2.804	0.584 ± 0.373	0.307
Thickness change 2 mm apically to the lingual GM	1.123 ± 2.96	4.093 ± 4.656	0.126
Vertical difference at the lingual GM	0.302 ± 0.304	0.278 ± 0.171	0.844

## Discussion

The results from the present randomized clinical trial have shown that both types of implants were associated with stable marginal bone levels and an improvement in the soft tissue contour. Both groups showed minimal bone loss 1 year after loading (0.16 mm in the test group and 0.45 mm in the control group). However, no statistically significant differences were detected when comparing the convergent and the straight implant neck.

These results are comparable to those published by Agustín-Panadero et al. 2019 [12], who compared MBL changes between bone-level implants placed at the crestal level or implants with a convergent transmucosal neck placed supra-crestally. At 24 months, the straight transmucosal neck group showed a mean bone loss of 0.61 ± 0.60 and the test implant of 0.24 ± 0.22 mm. The same research group [8] compared the tissue-level convergent neck implant with a divergent tissue-level implant. Again, they found less marginal bone loss around implants with a convergent transmucosal neck (0.29 ± 0.34 mm) than around the standard tissue-level implant (0.60 ± 0.63 mm). One possible explanation to understand why the convergent neck design is associated with more stable MBLs may be explained by the fact that this design creates less compression in the cortical bone and thus minimizes bone remodeling [4].

The convergent transmucosal neck presents a similar philosophy to the platform switching concept. The idea is to give the soft tissue a greater horizontal space and to shift coronally the implant-abutment connection, which at the same time could prevent bone remodeling by providing a better sealing [25]. Under this scenario, there was a study comparing MBLs changes 3 years after loading between standard tissue-level implants and bone-level implants with or without platform switching. The results showed that at the end of the study, MBLs remained stable only in the bone-level implants with platform switching [13]. Similar results have been reported in another 3-year study concluding that platform switching restorations showed a significant effect in the stability of marginal bone levels compared to those with platform matching (bone loss of 0.28 ± 0.56 mm and 0.68 ± 0.64, respectively) [26].

It should be noted that peri-implantitis developed fast and it was greater in the test group (3 implants in the test and 1 implant in the control). This is in agreement with the study by Derks et al. that showed that the onset of peri-implantitis occurred early [27]. This early onset could be explained by the fact that periodontitis patients were included in the study or because annual follow-up programs may not be enough to prevent the onset of peri-implantitis [28].

In the present study, we have observed a greater increase in soft tissue contour thickness around the convergent transmucosal neck (1.96 ± 2.69 mm) than in the conventional bone-level implant (0.65 ± 0.42 mm). However, this difference has not been statistically significant. Similar results around the convergent transmucosal neck have been reported in a recent case series, showing that these implants, together with crowns using the B.O.P.T. philosophy, experienced a significant increase in soft tissue thickness, both at the buccal (0.56 ± 0.46 mm) and at the lingual (0.33 ± 0.45 mm) surfaces 10 months after prosthetic loading [29]. Moreover, it should be noted that similar results could be achieved with a two-piece implant with a convergent abutment tightened at the moment of surgery.

Different esthetic parameters have been reported when using implants with a convergent transmucosal neck. In the prospective study published by Patri et al. 2017 [30], the pink esthetic score (PES) [14] and the papilla index [31] were evaluated as clinical and esthetic parameters of the peri-implant soft tissues 1 year after loading. As in the present study, there was a significant improvement in the appearance of the soft tissues, and the PES moved from 7.30 ± 2.80 at baseline to 11.95 ± 1.04 1 year after loading. Also, there was an improvement at the interproximal area, and the papilla index moved from 1.48 ± 0.59 at the mesial papilla and 1.59 ± 0.50 at the distal papilla at 6 months to 1.92 ± 0.49 at the mesial papilla and 2.07 ± 0.52 at the distal papilla at the 12-month follow-up. Nevertheless, since the outcome evaluated in the study [30] deferred from the ones reported in the present investigation, direct comparisons are not possible.

Several studies have shown that the most frequent pattern was the apical migration of the mucosal margin [18, 19], which is opposite to what has been shown in the present study. In all these studies, the final restorations were made on abutments with a horizontal finishing line. In the case of the present study, a BOPT restorative philosophy was used in the test group by using abutments without a finishing line that continue with the convergent transmucosal neck of the implants. There are no other studies comparing both restorative philosophies on implants, but there are studies that have compared the stability of soft tissues in tooth-supported prostheses following both philosophies, showing that prosthesis without finishing line following the BOPT philosophy provided greater soft tissue stability as compared to the horizontal finishing line preparation [32].

There is a great heterogeneity in the parameters and methods used to evaluate the esthetics and the volume of the peri-implant soft tissues [33]. Hence, there is a need to develop objective, reproducible, and non-invasive evaluation methods. One of the non-invasive methods that has been evaluated in the literature to monitor soft tissue thickness and its changes is ultrasound [34]. Although it is an accurate tool to assess the real thickness of the soft tissue [35] that may be interesting for research purposes, it may be not efficient for the daily practice. Today, digital measurement methods have been developed by using intraoral scanners and best-fit software. The OraCheck software (Cyfex, Switzerland) used in the present study is a valid method with a mean measurement error of 1.8 ± 1.1 µm and a maximum error of 5 µm [21, 23]. It is a tool that has been used in multiple studies to measure linear distances and volume changes [8, 21–23]. The methodology is based on the superposition of digital models. In the present study, longitudinal slices of the overlap between the digital model at loading and at 12 months were made at the level of the restoration and horizontal measurements were taken to assess the difference in contour thickness, and vertical landmarks were established to determine the position of the mucosal level. This methodology has been validated in previous studies [18, 19]. However, it has the main limitation that although it is very accurate to monitor contours, it has not the possibility to discern how much of the measurement is related to hard or soft tissues. Also, it is important to acknowledge that the small sample size evaluated in the present investigation may have influenced the absence of statistically significance for any of the comparisons. Moreover, the short follow-up may mask the role of the convergent transmucosal neck on providing a greater space for the soft tissue and its impact on peri-implant tissue behavior in the long term. It should be acknowledged that the presence or not of adjacent teeth to the studied implant may play a role in the support of soft tissue and the 3D dimensions; however, due to the small sample size, we were not able to evaluate the differences between the different clinical situations. Even though we have tried to include patients with minimal tissue thickness in terms of keratinized gingiva and buccal and lingual bone thickness, we cannot assure that they may not have acted as confounding factors in bone loss or in the final soft tissue contour. In fact, gingival phenotype was not taken into consideration. There are some studies concluding that implants placed with an initial peri-implant vertical mucosal thickness greater than 2 mm resulted on a minimum degree of crestal bone remodeling after 1-year follow-up [36, 37]. In contrast, the study of Spinato et al. found that platform-switching implants placed at a crestal level with short healing abutments demonstrated twice bone level loss than with long healing abutments irrespectively of initial mucosal thickness [38]. Another confounding factor could be the depth of the implant shoulder in relation to the initial soft tissue thickness [39, 40]. It should be noted that a 12-month period may not be enough to distinguish between physiological and pathological bone loss, and further studies with a long-term follow-up are needed to evaluate the impact of the implant neck configuration on bone resorption during the early and late stages of healing.

## Conclusions

Taking into consideration the limitations found in the present study, it can be concluded that both implant designs were associated with stable MBLs and soft tissue parameters 1 year after loading. New prospective clinical trials with a greater sample sizes and longer follow-ups are necessary to evaluate the effect of implants with a convergent transmucosal neck and restorations following the B.O.P.T. technique.

## Data Availability

The research data is available through contact with the corresponding author and is registered at the Complutense University of Madrid.
